# A rare intraoperative spinal cord injury caused by thoracic 8 nerve root interruption during posterior vertebral column resection surgery for severe congenital kyphoscoliosis: a case report

**DOI:** 10.1186/s12883-020-01785-2

**Published:** 2020-05-21

**Authors:** Shujie Wang, Zhifu Ren, Zhen Yang, Jianguo Zhang

**Affiliations:** 1 Department of Orthopedics, Peking Union Medical College Hospital, Chinese Academy of Medical Sciences & Peking Union Medical College, 1 Shuai Fu Yuan, Beijing, 100730 P. R. China; 2 Department of Spine Surgery, Weifang Municipal Traditional Chinese Hospital, Weifang, Shandong 261041 P. R. China; 3 Department of Orthopedics, Guizhou Provincial People’s Hospital, Guiyang, Guizhou 550002 P. R. China

**Keywords:** Severe congenital kyphoscoliosis (CKS), Posterior vertebral column resection (PVCR), Intraoperative neurological monitoring (IOM), Thoracic 8 nerve root

## Abstract

**Background:**

To our knowledge, the exposed nerve roots in thoracic spine are usually sacrificed to facilitate osteotomy during posterior vertebral column resection (PVCR) for severe spinal deformity. Currently we report a case with severe spine deformity in which intraoperative neurological monitoring (IOM) loss after interrupting T8 nerve root finally led to spinal cord injury during PVCR surgery.

**Case presentation:**

The patient was a 14-year-old female with severe congenital kyphoscoliosis (CKS) without preoperative neurologic deficits. The IOM events (MEP loss and SSEP latency prolong) were showed when T8 nerve root at concave side was interrupted. And then we reduce the scope of osteotomy to control bleeding, raised blood pressure (MAP, 65–80) to increase blood supply for spinal cord, placed the bilateral rod to stabilized the spinal cord, used the methylprednisolone, explored the presence or absence of spinal cord compression, and prepared to change the surgical plan from PVCR to PSO. After that the IOM signals partial recovered from the lowest point. Postoperatively the patients showed transient motor function deficits of left lower limbs weak without somatosensory deficits, and come back to preoperative status 6 months later.

**Conclusions:**

Interrupting the thoracic spine nerve root is danger to trigger the spinal cord injury during PVCR procedure of severe CKS. That probably because the increasing tension of contralateral anterior horn area of spinal cord via the nerve root pulling.

## Background

Posterior vertebral column resection (PVCR) has become a useful technique for treatment of severe and rigid spinal deformity; meanwhile it is one of biggest spinal surgeries and at higher risk of excessive blood loss and neurological deficits [1–4]. When surgeons were performing an osteotomy at the thoracic region, the nerve root of the corresponding section was often sacrificed. But Lenke et al. recommended placing temporary vascular clamps on the root sleeve for 5 to 10 min while checking the spinal cord monitoring to make certain no changes in the potentials are seen before ligation [5]. Until now there are few studies reporting the IOM events during the interrupting nerve root in PVCR procedure. Therefore, in the current study, we present a case of IOM event caused by interrupting T8 nerve root during PVCR for severe CKS.

## Case presentation

The patient included in this study provided written informed consent in accordance with the principles of the Declaration of Helsinki and the study was approved by Ethics Committee. And all methods were carried out in accordance with relevant guidelines and regulations.

**Fig. 1:**
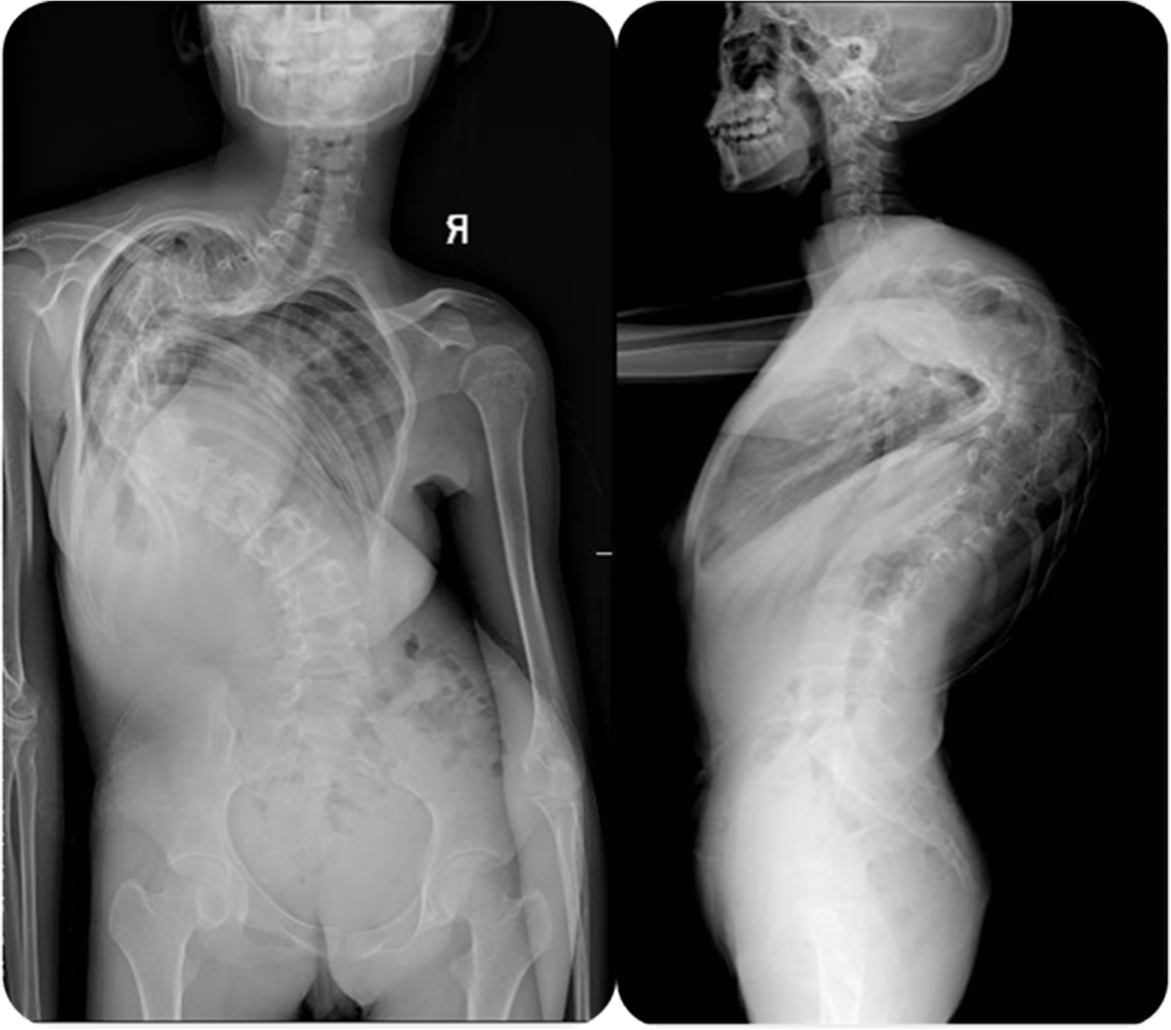
Preoperative standing, PA and lateral radiographs

**Fig. 2:**
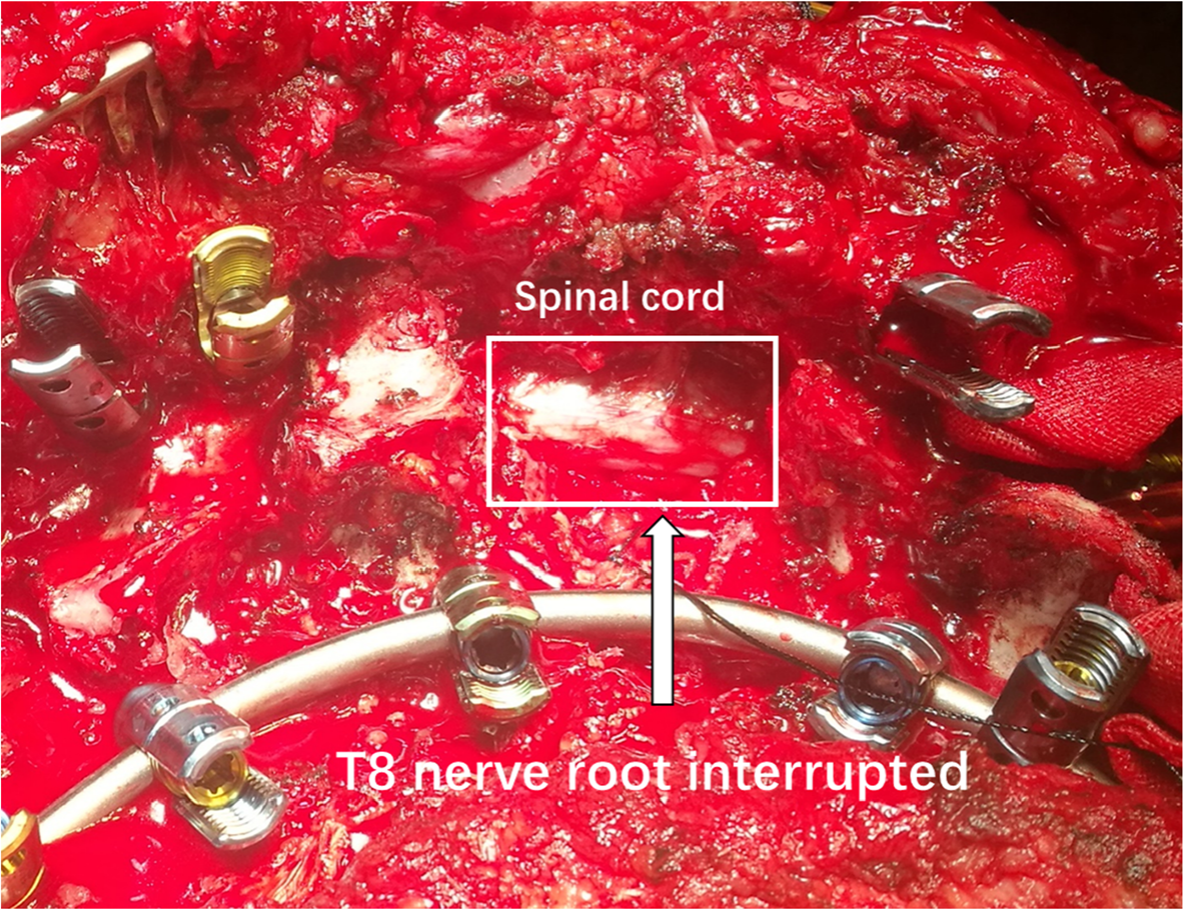
The exposed spinal cord and interrupted nerve root (T8 level) with a wide laminectomy and foraminotomy

**Fig. 3:**
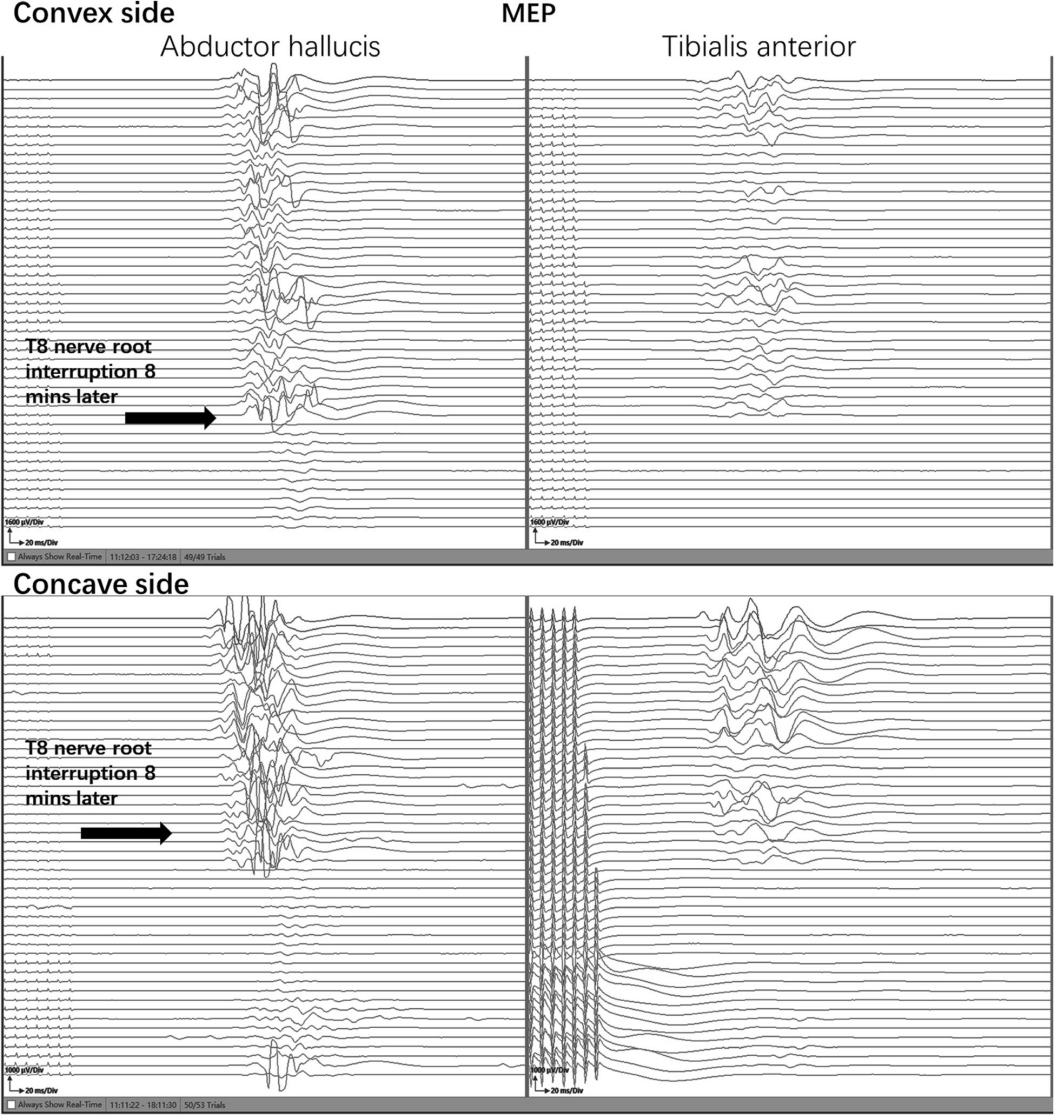
The whole process of interoperative MEP monitoring on both sides

**Fig. 4:**
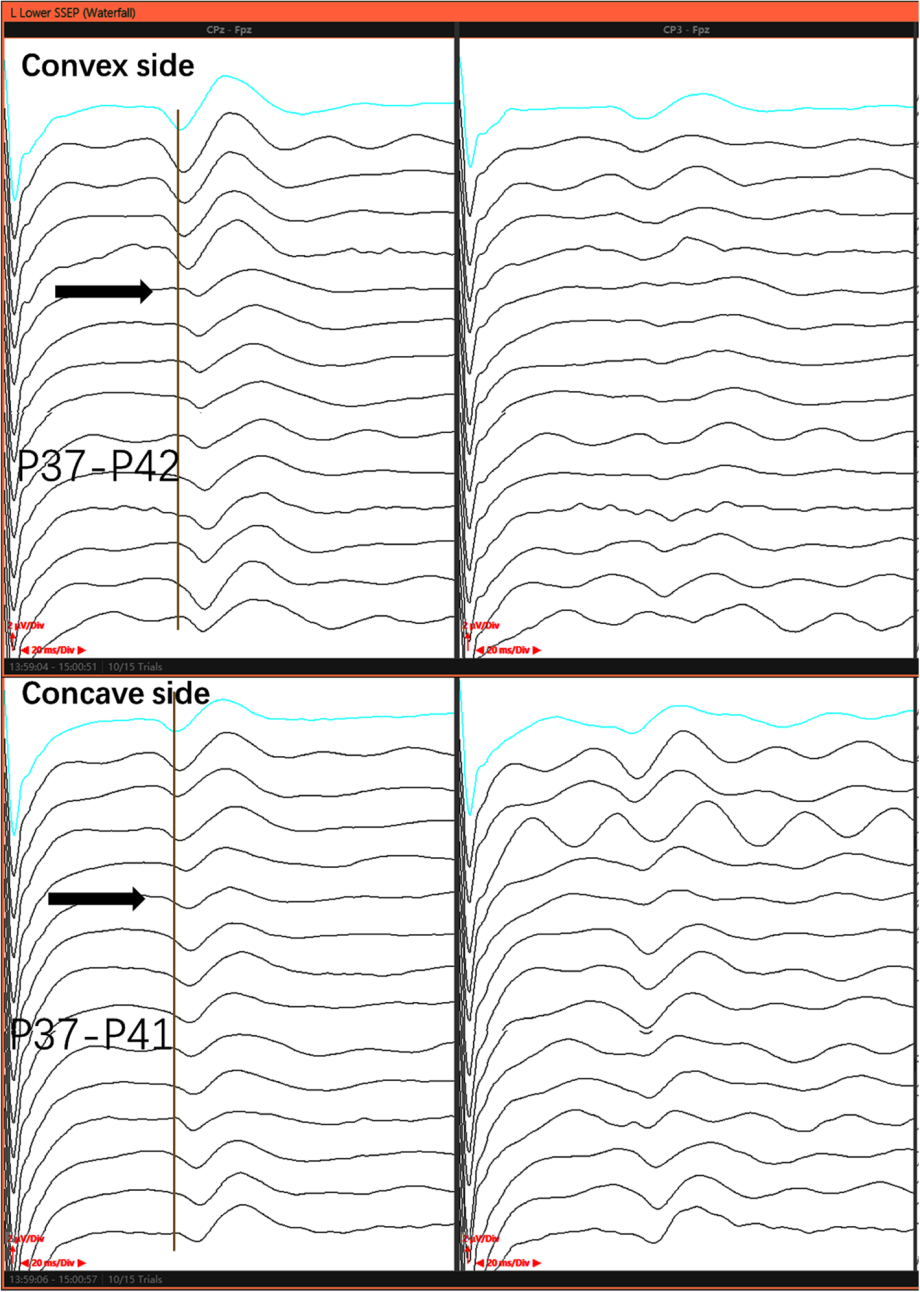
The whole process of interoperative SSEP monitoring on both sides

A 14-year-old female with severe congenital kyphoscoliosis (CKS) without preoperative neurologic deficits (Fig. 1). We were going to perform posterior T8 vertebral column resection (PVCR), correction, instrumentation from T2 to L3 and bone graft fusion. The pedicle screw insertion was extremely difficult in this case due to her complicated congenital spinal anomalies. The spinal cord and nerve root (T8 level) (Fig. 2, white arrow) were exposed with a wide laminectomy and bilateral foraminotomies. After a temporary rod was placed, the T8 nerve root at concave side was interrupted in order to sufficient exposure and osteotomy. About 8 min later the IOM (MEP) showed significant and instantaneous loss on the convex side first (Fig. 3), and then secondarily presented MEP loss on concave side. At the same time SSEP were also showing the prolonged latency and decreased amplitude (Fig. 4). After ruling out the system and anesthesia factors we thought this IOM event was related to the interruption of the T8 nerve root. The patient consent in this study was obtained.

And then we reduced the scope of osteotomy to control bleeding, raised blood pressure (mean arterial pressure, MAP from 65 to 80) to increase blood supply for spinal cord, placed the bilateral rod to stabilized the spinal cord, used the methylprednisolone, explored the presence or absence of spinal cord compression, and prepared to change the surgical plan from PVCR to pedicle subtraction osteotomy (PSO). After those interventions the IOM signals partial recovered from the lowest point.

Postoperatively the patients showed transient motor function deficit of left lower limb weak without somatosensory deficits, and then came back gradually to preoperative status 6 months later (Table 1).

**Table 1:** Postoperative motor function status of both lower limbs

Muscle strength (Convex side)	Proximal	Distal	Muscle strength (Concave side)	Proximal	Distal
**The first day**	0	I	**The first day**	IV	IV
**One week**	II	III	**One week**	V	V
**One months**	II	III	**One months**	V	V
**Two months**	III	IV	**Two months**	V	V
**Three months**	IV	IV	**Three months**	V	V
**Six months**	V	V	**Six months**	V	V

## Discussion and conclusions

In this case, the IOM events were happened 8 min later after T8 nerve root interruption. So, the unresponsive IOM alerts during surgery were difficult to find the point to point reason, fortunately the surgeon soon recognized that the nerve root interruption during osteotomy will potentially increase the tension from the other side on the ventral horn of spinal cord. And then we rapidly reduced the scope of osteotomy to control bleeding, raised blood pressure (MAP, 65–80) to increase blood supply for spinal cord [6], placed the bilateral rod to stabilized the spinal cord, used the methylprednisolone, explored the presence or absence of spinal cord compression, and prepared to change the surgical plan from PVCR to PSO. After those series of interventions, the IOM signals partial recovery from the lowest point. Therefore, after the significant high-risk surgical points it was very necessary to continue observing the IOM signals for a few minutes, and appropriate timely interventions were very useful in restoring the signals [7–9].

**Fig. 5:**
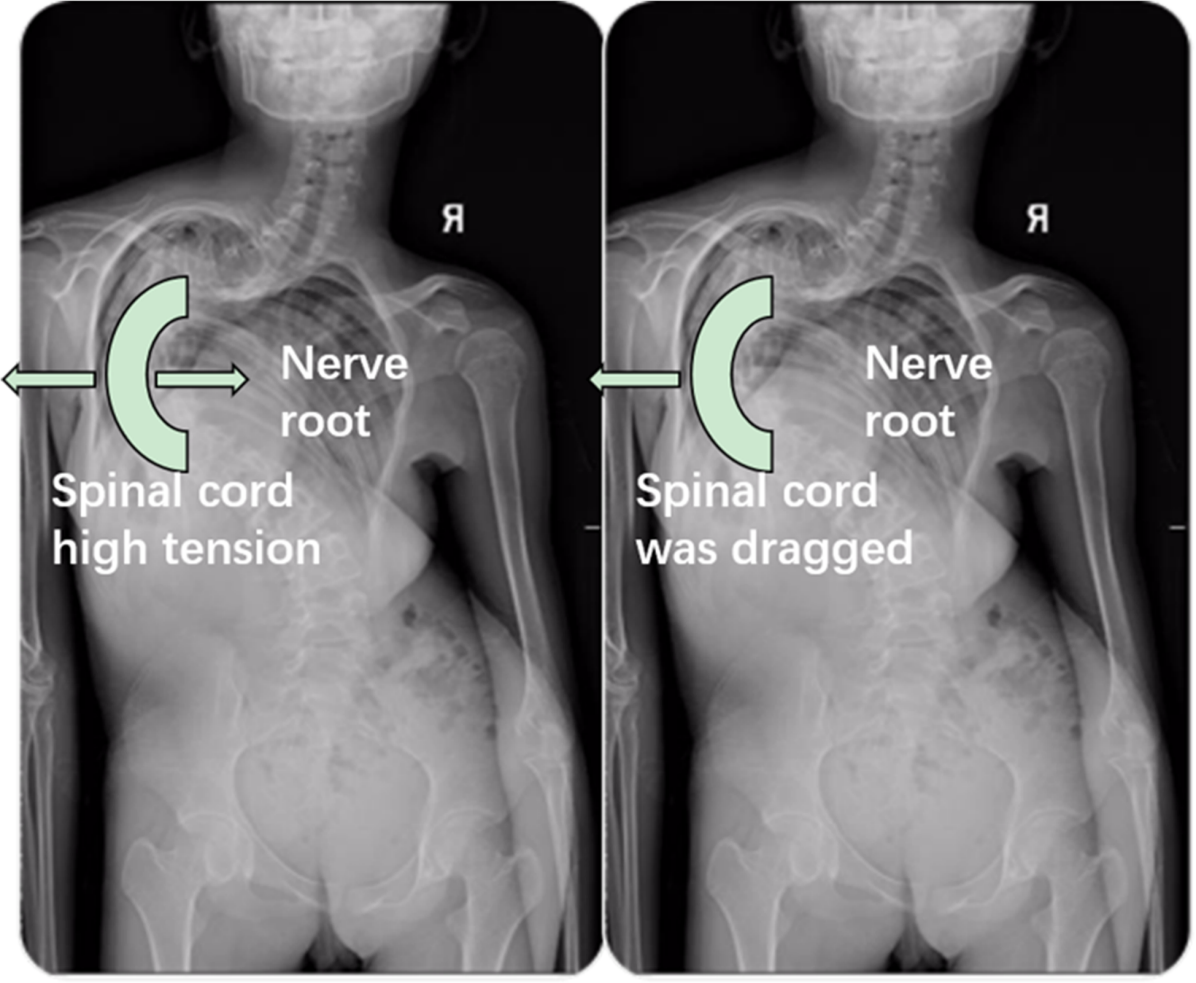
The schematic diagram of spinal cord tension change via unequally nerve root pull

According to many previous reports and our experience, among thoracic spine PVCR procedure interruption of several intercostal vessels and nerve roots was usually inevitable and low spinal cord injury risk [10–12]. However, in this case, we certainly found the strong correlation between the T8 nerve root interruption and concomitant IOM events. The reason was probably the increasing ventral horn tension of spinal cord via unequally nerve root pull (Fig. 5). Severe spinal CKS or scoliosis was a very critical inducement factor in this situation. Although most cases could not present spinal cord injury because of interrupting the thoracic spine nerve roots, we still treated that as a high-risk surgical procedure afterwards and needed to be looked at serious.

This case suggested that during patients with severe scoliosis originally the spinal cord tension at the apex was relatively high, and suddenly interrupting the thoracic spine nerve root on concave side was danger to trigger the stretching force increase on the other side of spinal cord by nerve root drag. So, we mightily agreed with the experience from Lenke et al. that temporary vascular clamps should be placed on the root sleeve for several minutes while paying closely attention to the IOM signal to make certain no changes in the potentials are seen before ligation [5].

There are also some points should be noted to this case. On one hand, we did not record the preoperative IOM baseline especially SEP data. Although we performed clinical nervous system physical examination in detail, some hidden electrophysiological level abnormalities were still difficult to be found. So, the evaluation of preoperative spinal cord injury risk was not comprehensive enough. On the other hand, postoperative immediately imaging data especially MRI were absent for this case, which created that ischemic spinal cord injury could not be ruled out completely. According to our experience, the T4-T9 spinal cord segment was most likely to occurring cord ischemia on account of interrupting nerve root accompanying vessels. But the following 2 diagnostic evidence probably can help to prove our hypothesis.

On one hand, we strictly followed up the patient’s neurological function changes and found that was gradually recovery post operation. The patient could try to get out of bed and walk 1 month after surgery, and then the neurological function came back to preoperative status completely 6 months post operation. According to the neurological function’s recovery the spinal cord injury was more like to mechanical injury of nerve pulling rather than ischemic injury. Because an ischemic insult can be more likely to lead to longer lasting or permanent injury than mechanical injury to the spinal cord [13–15].

On the other hand, even more importantly, if the spinal cord injury is due to ischemic or other factors, both of the IOM data and postoperative neurological symptom should be happening in the ipsilateral rather than contralateral side. Nevertheless, according to the IOM data and postoperative neurological symptom, the side of spinal cord injury was on the convex side (left), which exactly adhere to our hypothesis that the neural jury is probably because increasing tension of contralateral nerve root.

Interrupting the thoracic spine nerve root is danger to trigger the spinal cord injury during PVCR procedure of severe CKS. That probably because the increasing tension of contralateral anterior horn area of spinal cord via the nerve root pulling.

## Data Availability

All data generated or analyzed during this study are included in this published article.

## Abbreviations

IOM: Intraoperative neurological monitoring
PVCR: Posterior vertebral column resection
PSO: Pedicle subtraction osteotomy
CKS: Congenital kyphoscoliosis
MEP: Motor evoked potential
SEP: Somatosensory evoked potential
